# Lipid polymorphism of plant thylakoid membranes. The dynamic exchange model – facts and hypotheses

**DOI:** 10.1111/ppl.70230

**Published:** 2025-04-19

**Authors:** Győző GARAB, Kinga BÖDE, Ondřej DLOUHÝ, Zoltán NÁSZTOR, Václav KARLICKÝ, András DÉR, Vladimír ŠPUNDA

**Affiliations:** ^1^ Department of Physics, Faculty of Science University of Ostrava Ostrava Czech Republic; ^2^ Institute of Plant Biology HUN‐REN Biological Research Centre Szeged Hungary; ^3^ Institute of Biophysics HUN‐REN Biological Research Centre Szeged Hungary

## Abstract

The light reactions of oxygenic photosynthesis are performed by protein complexes embedded in the lipid bilayer of thylakoid membranes (TMs). Bilayers provide optimal conditions for the build‐up of the proton motive force (pmf) and ATP synthesis. However, functional plant TMs, besides the bilayer, contain an inverted hexagonal (H_II_) phase and isotropic phases, a lipid polymorphism due to their major, non‐bilayer lipid species, monogalactosyldiacylglycerol (MGDG). The lipid phase behavior of TMs is explained within the framework of the Dynamic Exchange Model (DEM), an extension of the fluid‐mosaic model. DEM portrays the bilayer phase as inclusions between photosynthetic supercomplexes – characterized by compromised membrane impermeability and restricted sizes inflicted by the segregation propensity of lipid molecules, safe‐guarding the high protein density of TMs. Isotropic phases mediate membrane fusions and are associated with the lumenal lipocalin‐like enzyme, violaxanthin de‐epoxidase. Stromal‐side proteins surrounded by lipids give rise to the H_II_ phase. These features instigate experimentally testable hypotheses: (i) non‐bilayer phases mediate functional sub‐compartmentalization of plant chloroplasts – a quasi‐autonomous energization and ATP synthesis of each granum‐stroma TM assembly; and (ii) the generation and utilization of pmf depend on hydrated protein networks and proton‐conducting pathways along membrane surfaces – rather than on strict impermeability of the bilayer.

## 1 INTRODUCTION

The ultrastructure of chloroplasts and its dynamic features set the conditions for the operation of the photosynthetic machinery under a broad range of environmental and metabolic conditions. The structural and functional plasticity of thylakoid membranes (TMs) – as will be emphasized in this review – are largely determined by their lipid phase behavior. Recent data on lipid polymorphism of TMs, besides leading to an extension of the ‘standard’ fluid mosaic membrane model (Singer and Nicolson 1972), may give new insights into the mechanism of light‐energy conversion in plant chloroplasts.

TMs in all oxygenic photosynthetic organisms “appear as an ensemble of flat sacs with highly curved edges” (Perez‐Boerema et al., 2024). In plant chloroplasts, they reside in the aqueous matrix, called the stroma. They are constituted of grana, stacked piles of cylindrical layers of TMs with 400–600 nm in diameter, and stroma lamellae, unstacked TMs, which are helically wound around the grana and are connected to them through narrow slits (Mustárdy and Garab 2003). Neighboring granum‐stroma TM assemblies are also coupled to each other (Bussi et al., 2019). By this means, the intricate 3D TM network system forms a single continuous membrane which encloses a contiguous interior aqueous phase, the lumen.

Plant TMs accommodate the two photosystems, PSII and PSI, along with their light‐harvesting antenna complexes, LHCII and LHCI, respectively. The stacked regions are enriched in PSII‐LHCII supercomplexes, which, under certain conditions, may form large, often semi‐crystalline arrays (Dekker and Boekema 2005; Wietrzynski et al., 2024). These supercomplexes display long‐range chiral order, both in vitro and in vivo (Garab 2014). PSI‐LHCI and the ATP synthase are located in the stroma lamellae. Additional components of the light‐energy converting machinery, the cytochrome *b*_6_ *f* complex (cyt *b*_6_ *f*) and the plastoquinone molecules are, respectively, embedded and shuttling in the lipid bilayer. Further constituents of the linear electron transport chain – the oxygen evolving complex, plastocyanin, ferredoxin and the ferredoxin:NADP oxidoreductase – are found in the aqueous phases of the TMs. Water‐soluble proteins also include the lumenal and stromal‐side lipocalin‐like photoprotective enzymes of the xanthophyll cycle, violaxanthin de‐epoxidase (VDE) and zeaxanthin epoxidase (ZEP) Goss and Latowski 2020). In general, the bilayer is densely packed by membrane proteins and the inter‐thylakoidal and stromal space and the lumen are also crowded with proteins (Kana et al., 2009; Kirchhoff et al., 2011; Gollan et al., 2021; Farci and Schröder 2023).

The operation of the electron transport system and the associated proton transport generate an energized state, proton‐motive force (pmf) or Δμ_H_^+^, consisting of a transmembrane electric potential gradient and a ΔpH. The generation of pmf and its chemiosmotic utilization (Mitchell, 1966) are based on the capability of TMs of insulating the inner aqueous phase from the outer aqueous phase. This is warranted by organizing the bulk lipid molecules into bilayer structures, which is the basis of the ‘standard’ fluid‐mosaic membrane model (Singer and Nicolson 1972). Lipid bilayers display low permeability to water and most water‐soluble molecules and to ions, and in particular protons (Miyamoto and Thompson 1967, Deamer and Bramhall 1986).

However, the organization of TM lipids into bilayer structures is not straightforward because only about half of the TM lipids – digalactosyldiacylglycerol (~25–30%), sulfoquinovosyldiacylglycerol (~10–15%) and phosphatidylglycerol (PG, ~10–15%) – are capable of spontaneously forming bilayers. The major lipid species of TMs, monogalactosyldiacylglycerol (MGDG, ~50%; Boudière et al., 2014), is a non‐bilayer lipid (Williams 1998). Non‐bilayer lipids, because of their conical shapes (Israelachvili 2011), are not capable of self‐assembling into bilayers. Lipid mixtures containing high concentrations of lipids with non‐cylindrical geometry tend to form non‐bilayer structures, such as inverted hexagonal (H_II_) or cubic phases, or assemble into small micelles. A deeper conflict with the ‘standard’ model is, that functional plant TMs contain non‐bilayer lipid assemblies in large quantities (Krumova et al., 2008a, Garab et al., 2017, Garab et al., 2022).

The high abundance of MGDG and the co‐existence of bilayer and non‐bilayer structures in TMs pose perplexing questions regarding their roles in the mechanism of energy conversion. Furthermore, it is now well established that, beside the well‐known fact that about half of the lipids of the inner mitochondrial membranes (IMMs) are non‐bilayer lipids (phosphatidylethanolamine, ~34%; cardiolipin. ~18%; Harwood 1985), functional IMMs also contain non‐bilayer lipid phases (Gasanov et al., 2018). The polymorphic phase behavior of TMs and IMMs was recently reviewed (Garab et al., 2022), but the origin of different lipid phases in terms of structural entities remained elusive.

Data obtained in the past three years, on isolated plant TMs and sub‐chloroplast particles, now allow us to provide a model that locates the different lipid phases in the chloroplast ultrastructure. Here, we show that the required bilayer organization of TMs and the high abundance of MGDG and non‐bilayer lipid phases can be reconciled with each other – by taking into account physical mechanisms that deviate from the original form of the chemiosmotic theory. Further, we set experimentally testable hypotheses on the roles of lipid polymorphism in the energization of TMs and its utilization for ATP synthesis – in the spirit of John Green: “as you learn, you don't really get answers; you just get better questions”.

## 2 LIPID POLYMORPHISM OF THYLAKOID MEMBRANES AND SUB‐CHLOROPLAST PARTICLES

Observations in the 1980s showed that isolated TM lipid mixtures assemble into non‐bilayer structures and suggested the possibility that similar phases could be formed in TMs. However, it was concluded that „non‐bilayer configurations were difficult to reconcile with the need to maintain a stable semipermeable membrane system” (Gounaris et al., 1986). Indeed, the occurrence of H_II_ phase, as identified by freeze‐fracture electron microscopy (FF‐EM), was only rarely observed: it required special conditions, e.g. exposure of TMs to 40°C, addition of co‐solutes (cf. Williams 1998), days‐long storage at 5°C (Semenova 1999), or isolating TMs from low‐light grown plants (Kirchhoff et al., 2007).

**FIGURE 1:**
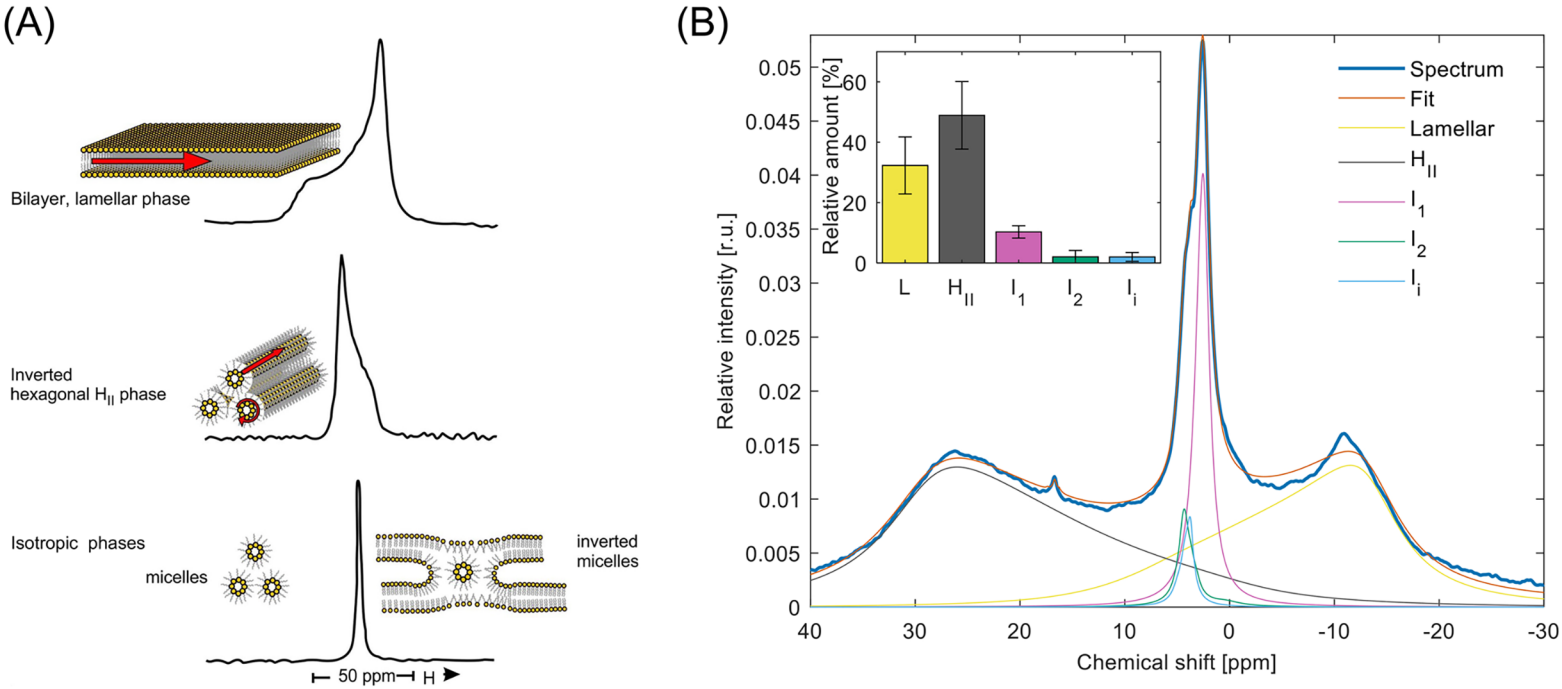
**Fingerprinting the lipid phase behavior of TMs by** ^**31**^ **P‐NMR spectroscopy**. (A) Bilayer and non‐bilayer lipid assemblies, indicating also the characteristic motions of lipid molecules (red arrows), and the corresponding ^31^P‐NMR spectra (based on Cullis and de Kruijff 1979). (B) ^31^P‐NMR spectrum of freshly isolated spinach thylakoid membranes (thick dark blue trace) and the fitted spectrum (orange curve) obtained after deconvolution of the spectral components. The area‐normalized spectrum represents the average obtained from 52 independent experiments performed at 5°C. The average Chl content of these samples was 8.3 ± 3.1 mg ml^−1^. Individual contributions of the lamellar (L, yellow), inverted hexagonal (H_II_, grey), and isotropic (I_1_, I_2_, and I_i_, pink, green and light blue, respectively) lipid phases were determined using the software DMfit (Massiot et al., 2002). Inset depicts the integrated areas of the deconvoluted component spectra, associated with the different lipid phases, relative to the overall integrated area; mean values ± SD, *n* = 52. (Note that the spike, a component of unknown origin with isotropic features, peaking at around 17 ppm, is not displayed in the figure.)

The first evidence that fully functional isolated intact TMs contain non‐bilayer lipid phases was presented by Krumova et al., (2008a) using ^31^P‐NMR spectroscopy (Figure 1A). Earlier, co‐existence of the bilayer and non‐bilayer phases was observed on lyophilized and TRIS‐treated membranes suspended in D_2_O (Harańczyk et al., 1995). In agreement with ^31^P‐NMR spectroscopy, time‐resolved fluorescence spectroscopy of Merocyanine‐540 stained TMs also revealed different physico‐chemical environments of the lipid molecules (Krumova et al., 2008b; Garab et al., 2017; Kotakis et al., 2018). For a short overview of different techniques that can be used to monitor lipid polymorphism and lipid phase transitions in native membranes and in lipid model systems, see Supplementary Information.

It has now been thoroughly documented that plant TMs, in addition to the bilayer (L, lamellar) phase, contain an H_II_ phase and at least two isotropic phases (Figure 1B).

Via employing ^31^P‐NMR spectroscopy on isolated granum and stroma TM preparations (Dlouhý et al., 2021a), it has also been demonstrated that the lipid polymorphism of TMs is not correlated with the lateral heterogeneity of proteins. It has also been clarified that no SAXS signature of the H_II_ phase could be discerned (Dlouhý et al., 2021b). The lack of long‐range order of lipid molecules was explained by the low lipid‐to‐protein molar ratios of the samples (0.25–0.30), i.e. by the high protein density of the sample.

**FIGURE 2:**
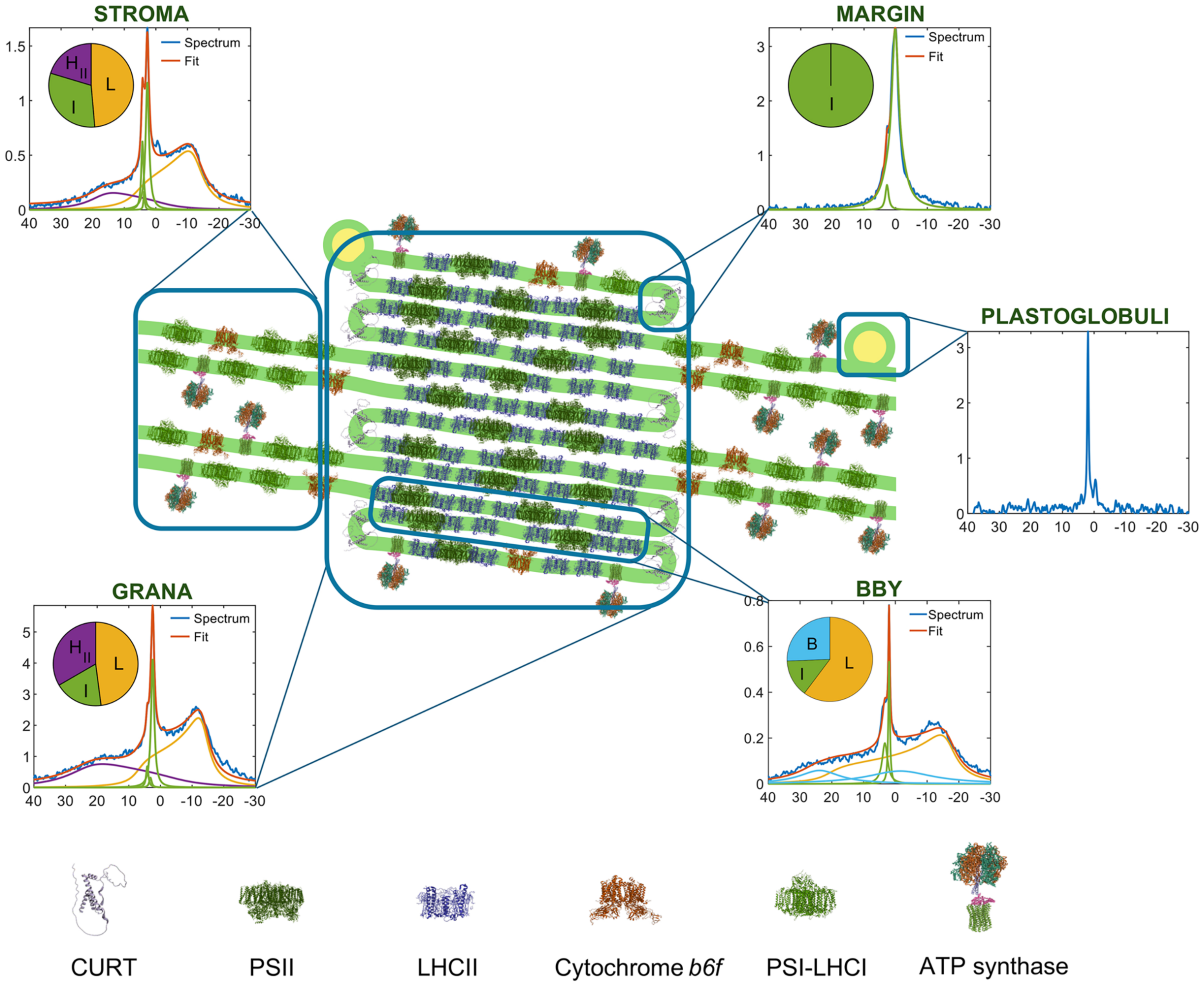
Schematic illustration of the TM ultrastructure and the ^31^P‐NMR spectra of different sub‐chloroplast particles. The relative amounts of the contributing lipid phases are displayed in insets (L, yellow – lamellar or bilayer phase; I, light green – sum of the sharp isotropic phases similar to those in TMs; H_II_, purple – inverted hexagonal phase; and B, light blue – sum of the two broad isotropic bands which are present only in BBY). For the membranous particles (grana, stroma, BBY), the counts are normalized for 10 mg ml^−1^ chlorophyll content and 6400 scans. For the MR and plastoglobuli preparations, the counts were normalized as described in (Böde et al., 2024a) and (Dlouhý et al., 2022), respectively. (Horizontal scales: chemical shift ‐ ppm, vertical scales: counts ‐ 10^4^, except for plastoglobuli ‐ 10^3^.) It is important to stress that the weak, phospholipase‐insensitive ^31^P‐NMR signal of plastoglobuli does not contribute to the lipid polymorphism of TMs (Dlouhý et al., 2022). Note that the granum and stroma TMs exhibit very similar polymorphisms, resembling that of intact TMs, showing that the PSII‐LHCII and PSI‐LHCI supercomplexes have no preferred lipid phases (Dlouhý et al., 2021a). The absence of H_II_ phase in BBY is consistent with the finding that this phase originates from association of lipid molecules with stroma‐exposed protein(s) or polypetide(s) – (Dlouhý et al., 2022; Böde et al., 2024a). The MR displays an intense isotropic phase (Böde et al., 2024b), probably due to the presence of loosely attached lipid molecules that surround the CURT proteins (PDBs: AF_AFA0A1D6K7J4F1, 9EVX, 6A2W, 2ZT9, 8J7A, 1QO1).

Recently, we also fingerprinted the lipid phase behavior of the stacked PSII membrane pairs (BBY particles; Böde et al., 2024a) and the marginal region (MR) of grana (Böde et al., 2024b). The ^31^P‐NMR spectra and ultrastructural positions of all sub‐chloroplast particles are displayed in Figure 2. This gallery also depicts the plastoglobuli (Dlouhý et al., 2022), which, however, contain only trace amounts of thylakoid lipids, and their proposed lipid exchange mechanism with TMs (Kirchhoff 2019) might thus be very slow.

It is evident that all membranous sub‐chloroplast particles exhibit intense L phases, but this phase is absent in MR, probably because of the lack of supercomplexes that would stabilize the bilayer (Böde et al., 2024b; Koochak et al., 2019). The H_II_ phase was absent in BBY and MR. In contrast, all samples exhibited sharp isotropic ^31^P‐NMR peaks with high amplitudes – indicating the participation of a large number of highly mobile lipid molecules in the corresponding structural entities.

## 3 LIPID PHASES IN DISTINCT DOMAINS OF THYLAKOID MEMBRANES

### 3.1 Lamellar phase

In perfect harmony with all that we know about the role of bilayer organization of the bulk lipid molecules, ^31^P‐NMR spectroscopy and electrochromic shift absorbance transient measurements performed under comparable conditions revealed close correlation between the weakening of the bilayer signature of TMs and the increased permeability of membranes (Ughy et al., 2019). Furthermore, the insulation capability of TMs was found to be very sensitive to Phospholipase A1 (PLA1; Dlouhý et al., 2022). The virtually immediate effect of this lipase on TMs was the acceleration of the decay of the electrochromic shift absorbance transient, an effect which resembled that of ionophores (Witt 1979). These data indicate an easy access of PLA1 to the bilayer and show that the energized state of TMs can be abolished by hydrolyzing the minor lipid PG. In other terms, by ‘punching a hole’ in the bilayer the TM becomes permeable to ions – corroborating the pivotal role of the bilayer organization of lipids in membrane energization. However, as it will be emphasized in paragraphs below, the bilayer of TMs must not be portrayed as large sheets embedding, or ‘scaffolds’ holding, the intrinsic proteins – but rather as a ‘patchwork’ of lipids that are enclosed between the densely packed supercomplexes. Further, as it will also be stressed below, the insulation capability of bilayers formed by TM lipids is inferior compared to bilayers composed of pure bilayer lipids.

### 3.2 Isotropic (I) phases

Regarding the I phases, we obtained important information by using WGL (wheat germ lipase), which selectively destroyed these phases of TMs – with no or very little effect on the L and H_II_ phases and virtually none on the structural and functional parameters of the photosynthetic machinery (Dlouhý et al., 2022). These data clearly indicated that the molecular assemblies responsible for the I phases were to be found in TM subdomains outside the protein‐rich bilayer area. I‐phase lipids were proposed to be involved in the fusions and junctions of TMs (Garab et al., 2017; Garab et al., 2022).

**FIGURE 3:**
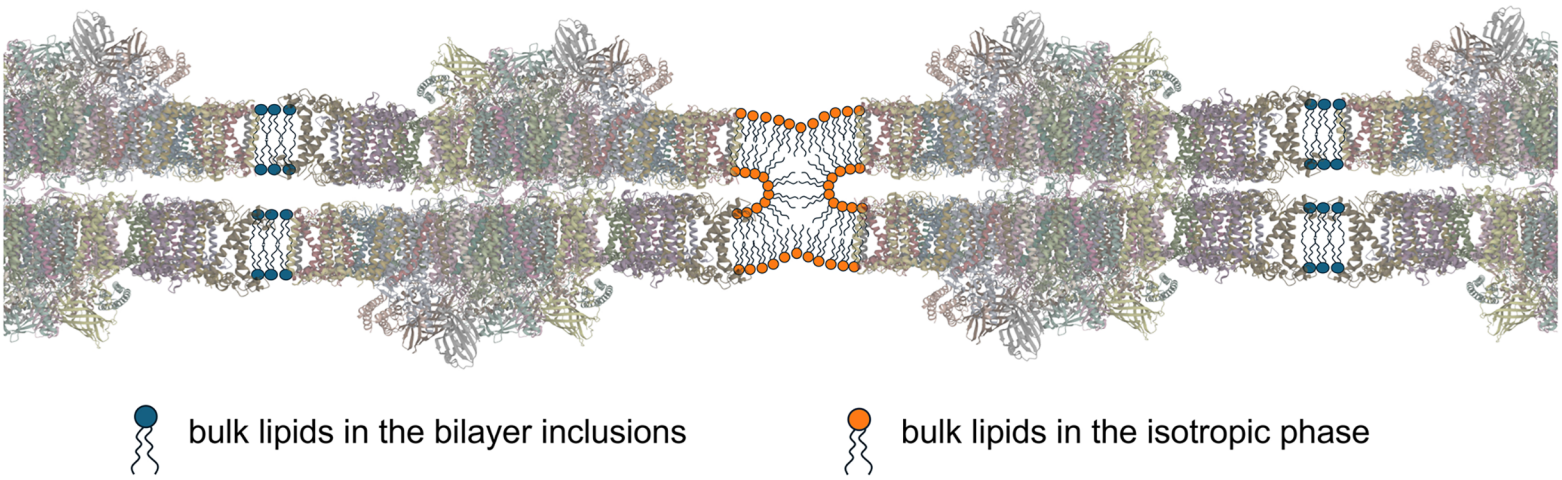
Schematic illustration of the involvement of the non‐bilayer, isotropic lipid phase in the lateral fusion of adjacent membrane pairs, BBY particles, enriched in PSII‐LHCII supercomplexes (PDB: 5XNL). Using the geometry reported by Boekema et al., (2000), the average area of a lipid “inclusion” between the supercomplexes is estimated to be ~130 nm^2^, which corresponds to 200–300 lipid molecules in one leaflet of the bilayer. The area occupied by a lipid molecule in bilayer structures varies between about 0.45 and 0.65 nm^2^ (cf; Hryc et al., 2022). The figure is modified after (Böde et al., 2024a). Similar restrictions for the area available for the bilayer phase would be obtained when using the mean center‐to‐center distance of 21.2 ± 3.1 nm between PSII supercomplexes (Wietrzynski et al., 2024), and the lateral dimensions of PSII‐LHCII (smallest, ~18 nm and largest, ~25 nm).

Experimental evidence for I‐phase‐mediated membrane fusion was provided by using BBY: WGL disassembled the large sheets of the laterally fused stacked PSII membrane pairs (Böde et al., 2024a; Figure 3). It might thus be inferred that I phase(s) – together with other key elements (see Section 4) – aid(s) the self‐assembly and plasticity of TMs. Chloroplasts contain regions with fusion and fission of membranes of marked dynamic features (Nevo et al., 2012). It is interesting to note here that I phases are present in all sub‐compartments of TMs and – as shown by electron microscopy and tomography data (Dlouhý et al., 2021b; Böde et al., 2024a; Böde et al., 2024b) – all of these samples form interwoven, multiply fused structures. In this context, de‐etiolation processes are of great interest. During those processes the paracrystalline tubular prolamellar body is transformed into a lamellar system (Solymosi and Schoefs 2010; Kowalewska et al., 2019; Sandoval‐Ibáñez et al., 2021; Liang et al., 2022; Wietrzynski et al., 2024).

It is to be noted here that the isotropic signal in MR might, in principle, originate from highly curved bilayers (Cullis and de Kruijff 1979). However, given the fact that intense I hases are present in all fractions, highly curved bilayers alone would not explain the occurrence of I phase (Krumova et al., 2008a). Also, the close correlation between the diminished insulation capability of membranes and the enhanced I phase(s), at the expense of the L phase, (Ughy et al., 2019; see 3.1) argue against well sealed bilayer structures in the MR.

**FIGURE 4:**
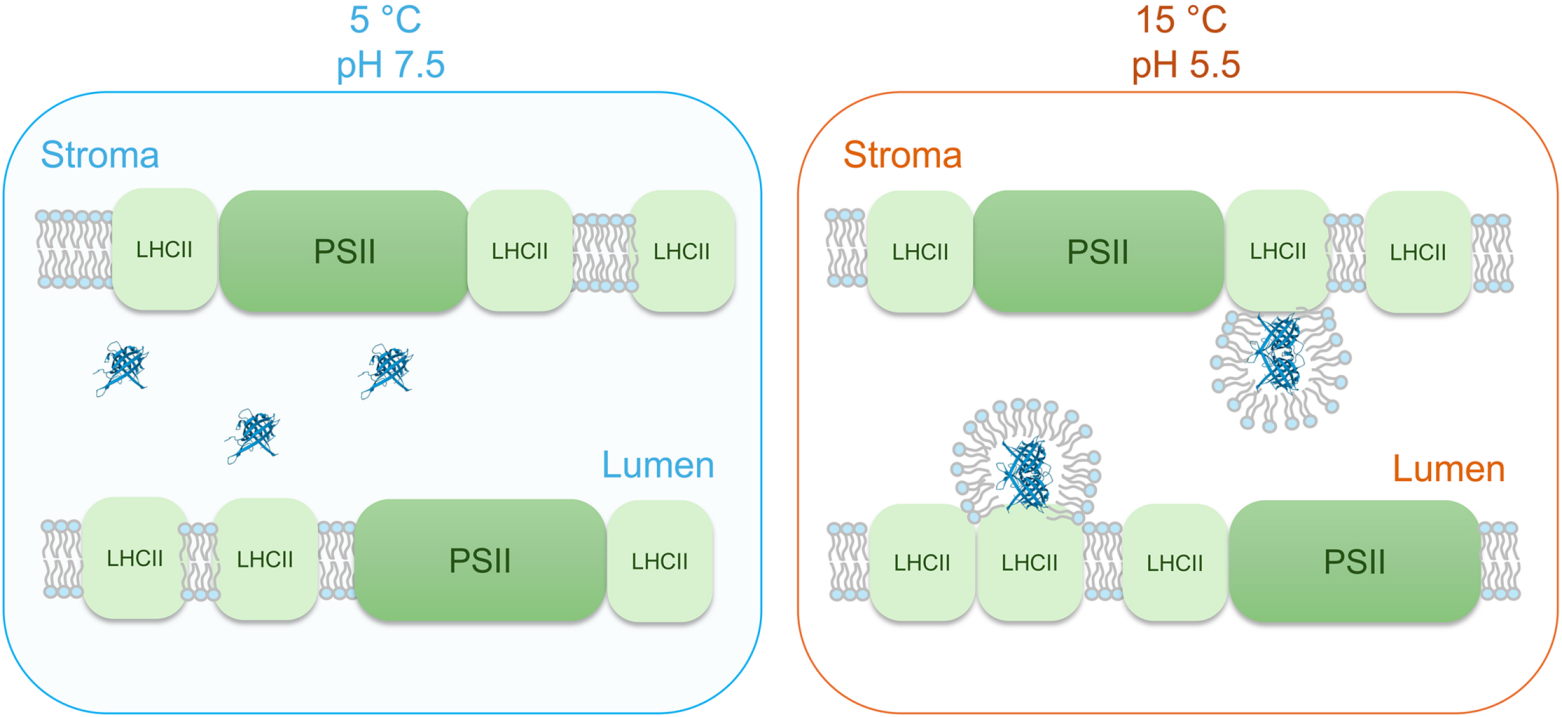
Proposed role of an isotropic, non‐bilayer lipid phase in VDE activity. The proposed model is based on the low‐pH and elevated temperature‐induced enhancement of an isotropic phase and the activity of VDE (Dlouhý et al., 2020). The envisioned association of I phase lipids with VDE dimers (PDB: 3CQN) is consistent with the earlier documented non‐bilayer lipid‐phase dependent activity of this lumenal photoprotective enzyme (Latowski et al., 2002, Goss and Latowski 2020).

In broad agreement with earlier findings on model systems (Latowski et al., 2002), an enhanced isotropic phase was also found to be associated with the activity of VDE (Dlouhý et al., 2020). This is envisioned to be formed in the lumenal phase (Figure 4). It is unclear whether or not ZEP on the stromal side forms an I phase similar to that of VDE in the lumen.

### 3.3 H_II_ phase

Unexpectedly, we found that Trypsin and Proteinase K selectively suppressed the H_II_ phase. At low and moderate concentrations, these proteinases destroyed the H_II_ phase with only minor or no effect on the other lipid phases (Dlouhý et al., 2022). This showed that, similarly to the I phases, the structural entity responsible for this phase is located in distinct subdomain(s) of TMs. Since, these proteases selectively hydrolyze peptide bonds exposed to the stromal side of TMs (Karnauchov et al., 1997), it has been concluded that H_II_ phase is given rise by a fraction of TM lipids surrounding or encapsulating stroma‐side protein(s) or polypeptide(s). Indeed, BBY particles, containing no stroma‐exposed proteins, possess no H_II_ phase. It is also interesting to note that the highly curved marginal region, which contains the Trypsin‐sensitive CURT1 protein, also does not give rise to this phase (Böde et al., 2024b). This renders our hypothesis (Dlouhý et al., 2022) about the participation of the CURT1 protein in the H_II_ phase highly unlikely. The identity of the protein(s) and/or polypeptide(s) that are associated with lipids displaying H_II_ phase remain to be determined.

## 4 THE DYNAMIC EXCHANGE MODEL OF THYLAKOID MEMBRANES

The polymorphic lipid phase behavior of TMs is interpreted within the frameworks of the Dynamic Exchange Model (DEM), an extension of the fluid‐mosaic membrane model. DEM postulates the coexistence of bilayer and non‐bilayer phases and their dynamic equilibrium, and a self‐regulatory mechanism that safeguards the high protein‐to‐lipid ratios in energy converting membranes (Garab et al., 2000; Garab et al., 2016). DEM has been based on: (i) the observation that MGDG can be forced by LHCII to form a bilayer membrane (Simidjiev et al., 2000), and (ii) the ability of lipid mixtures possessing high non‐bilayer propensity to segregate out from the bilayer (Seddon and Templer 1995). Garab et al., (2022) emphasized the importance of two additional attributes of TMs that largely determine the polymorphic phase behavior of TMs: (iii) both the inner and outer aqueous phases of TMs are fully packed with proteins (Kirchhoff et al., 2011), some of which, lipocalins or lipocalin‐like proteins, such as VDE, ZEP and LCNP, are capable of binding lipid molecules (Malnoë et al., 2018, Goss and Latowski 2020). Finally (iv), the self‐assembly and structural dynamics of TMs depend largely on the fusion of membranes and intermembrane exchange of lipids (Mustárdy et al., 2008, Bussi et al., 2019), which assume the formation of non‐lamellar lipid phases (Seddon and Templer 1995, Blumenthal et al., 2003). Non‐bilayer lipid phases have also been proposed to play important roles in the targeting, insertion, and assembly of proteins in plastid membranes (Bruce 1998; LaBrant et al., 2018).

Biogenesis and maintenance of thylakoid membranes have been shown to be governed by vesicle‐inducing protein in plastids 1 (VIPP1). These water‐soluble lipid binding proteins mediate contact between the thylakoids and chloroplast envelope and also play an important role in maintaining integrity under high light stress (Gupta et al., 2021). Under certain conditions these proteins, also called as IM30, have been shown to be capable of destabilizing the bilayer via pore formation (Junglas et al., 2022). External protein‐induced pore formation may contribute to the structural dynamics of TMs and may trigger membrane fusion. The formation of holes evidently brings about the emergence of non‐bilayer lipid phases. It is interesting to note that toroidal holes have recently been visualized in cardiac mitochondrial cristae near the ATP synthase (Nesterov et al., 2024).

Additionally, other proteins, such as the dynamin‐like protein CrFzl from the green algae *Chlamydomonas reinhardtii*, have been proposed to modulate membrane organization and stabilization through membrane remodeling (Findinier et al., 2019). Other candidates are the Plasma Membrane Fusion Protein (PMFP, AT5G42765) identified in *Arabidopsis thaliana* or the Tvp38/DedA Family Protein (TVPFP, AT1G22850), which show homology with other membrane contact site proteins (Friso et al., 2004; Niedermaier et al., 2020). It is equally important to emphasize the role of lipid transport between the endoplasmatic reticulum and the TMs, which is achieved by multiple mechanisms including membrane contact sites with specialized protein machinery (LaBrant et al., 2018).

In accordance with the predictions of DEM, the lipid phases of TMs have been shown to respond sensitively to changes in the physico‐chemical environment of the membranes, such as the pH and the ionic strength and osmolarity of the reaction medium. The lipid phases were particularly sensitive to elevated temperatures, which diminished and often eliminated the ^31^P‐NMR spectroscopy signature of the bilayer; at the same time, the contribution of resonances with isotropic signature increased (Krumova et al., 2008a, Dlouhý et al., 2020). However, it must be stressed that under comparable conditions the bilayer structure was preserved, albeit, with increasing the temperature, the energized state, parallel with the destabilization of the bilayer, relaxed faster (Dlouhý et al., 2020). Thus, vanishing the L phase at elevated but physiologically relevant temperatures is attributed to enhanced lipid exchange between the L and I phases. In general, it appears that non‐bilayer lipids lend high plasticity to TMs – in line with the notion that „the bilayer must not be too stable because that would tend to limit protein dynamics” (Bagatolli et al., 2010). However, as will be posited below, non‐bilayer lipid phases in TMs may play more fundamental roles in the conversion of light energy to chemical energy.

## 5 HYPOTHESES

### 5.1 Sub‐compartments, autonomy of the granum‐stroma assembly

We hypothesize that each individual granum‐stroma unit possesses a high degree of autonomy with regards to the generation of pmf and its utilization for ATP synthesis, and that this functional sub‐compartmentalization is assisted by one or several non‐bilayer lipid phases.

According to most textbooks, the aqueous phase of a chloroplast is divided by the TM into two compartments, and the local electric fields are “delocalized over the thylakoid membrane by ion redistribution within the inner and outer aqueous phase of the membrane” (Witt 1979). According to this picture, the pmf is generated between the two aqueous phases of TMs. However, this widely accepted simplified view should probably be modified by taking into account more recently established parameters of the ultrastructure and molecular organization of TMs.

**FIGURE 5:**
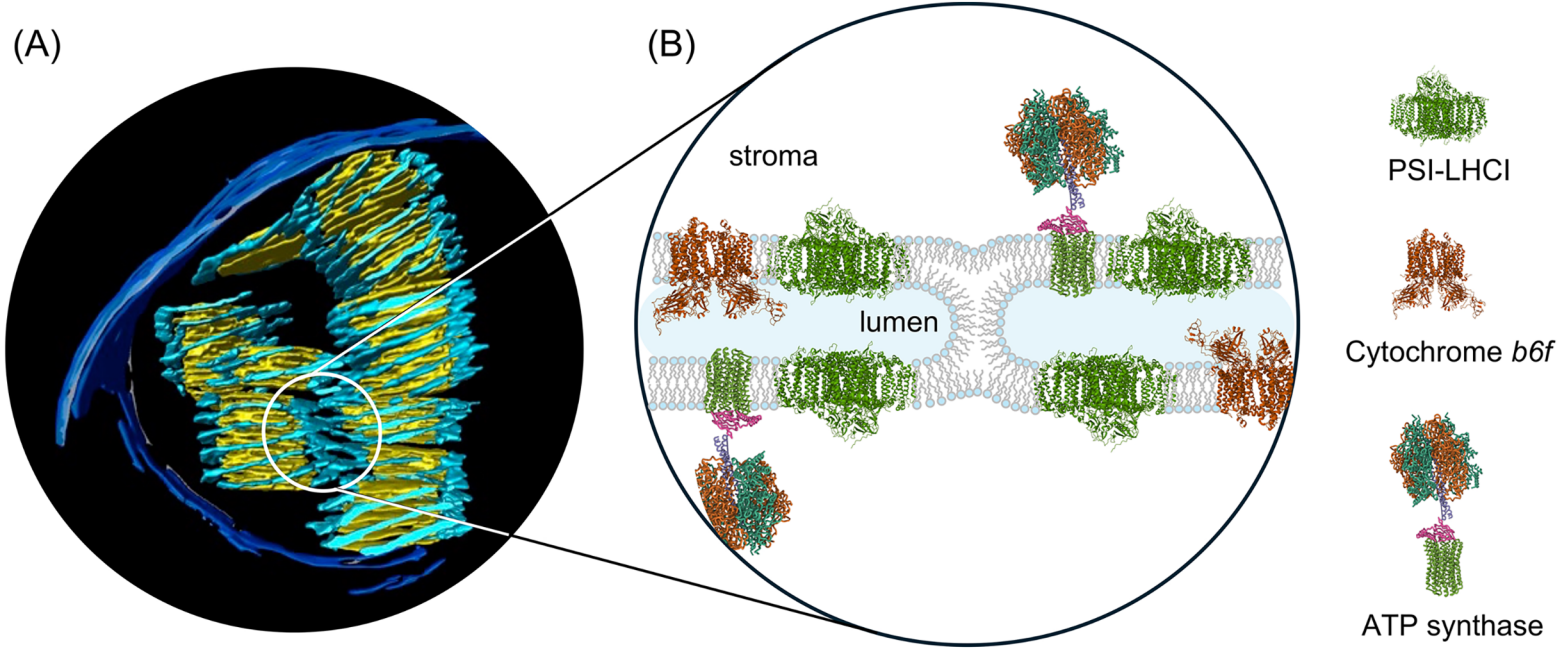
Sub‐compartmentalization of chloroplast TMs – energetic autonomy of granum‐stroma assemlies. (A) 3D model of the extensive, fusion‐rich vesicular network of thylakoid membrane reconstructed from electron microscopy tomography images; yellow: granum vesicles, light blue: stroma lamellae, blue: inner and outer envelope membranes (Bussi et al., 2019). (B) Proposed mechanism of the lumen‐lumen discontinuity involving a non‐bilayer lipid structure between adjacent stroma lamellae. For further explanation and arguments for the hypothesis of energetic autonomy of granum‐stroma units, see the main text. PDBs: 8J7A, 2ZT9, 1QO1).

First, we recall that each granum‐stroma assembly contains all components of the light‐energy‐converting machinery and that interconnections between the granum and stroma membranes and between the TM layers of grana (Mustárdy and Garab 2003) allow the formation of a delocalized uniform pmf in each unit. Thus, in principle, these units are capable of producing ATP with a high degree of autonomy. On the other hand, sharing pmf between adjacent granum‐stroma units may be sterically hindered. It has recently been clarified that the right‐handed helices of neighboring granum‐stroma assemblies are joined together via bifurcations of membranes and left‐handed helical surfaces (Bussi et al., 2019). These relatively narrow ‘bridges’ might constitute a barrier for ionic currents in the lumen – e.g. if these ‘bridges’ contain stalks or lipid assemblies similar to those involved in the lateral fusion of PSII membranes (Böde et al., 2024a). A possible scenario of disrupting the lumen‐lumen continuity by a non‐bilayer lipid phase is depicted in Figure 5.

Energetically, and to reach the threshold of pmf, autonomy of granum‐stroma units is evidently advantageous compared to sharing the energized state of each unit with all other granum‐stroma assemblies in a chloroplast. In this context, it is interesting to evoke the photophosphorylation autonomy of purple bacterial chromatophores (Altamura et al., 2021) and the energetic autonomy of individual cristae of mitochondria (Wolf et al., 2019).

### 5.2 Compromised membrane impermeability; the role of protein networks in utilizing pmf

Recently Fehér and coworkers (Fehér et al., 2023) compared the permeability of two bilayers using coarse‐grained molecular dynamics (MD) simulation: one, constructed from a TM lipid mixture and the other from DPPC (dipalmitoyl phosphatidylcholine). They found that the permeability of the bilayer composed of TM lipids was almost two orders of magnitude larger than that of the membrane formed by the typical bilayer lipid DPPC. Recent MD simulations using TM lipid mixtures have further revealed that MGDG promotes dynamic fluctuations in membrane thickness and spontaneous formation of inverted hexagonal phases and other, not well‐defined non‐bilayer structures (Fehér et al., 2025). These processes depend heavily on the concentration of MGDG as well as on the hydration state and temperature of the lipid assemblies. These findings are in harmony with the generally accepted view that biological membranes that contain non‐bilayer lipids are in a frustrated state and that “a membrane composed solely of lamellar lipids would be an optimum insulator, only non‐compatible with cell function, i.e., life” (Goñi 2014).

It is an intriguing question whether the inferior electrical insulation capability due to the presence of MGDG is simply an unavoidable consequence of the presence of non‐bilayer lipids requested for ‘secondary’ functions of TMs (such as the fusion of membranes, and lipocalins), or these lipids play a more fundamental role in the energy conversion. The answer to this question depends on the physical and molecular mechanisms of the energization of TMs.

The nature of the energized state and the mechanism of its utilization for ATP synthesis in energy‐converting biological membranes is long‐debated. Many authors proposed mechanisms deviating from or modifying the original form of the chemiosmotic theory of Mitchell (1966) (Williams 1979; Cherepanov et al., 2003; Dilley 2004; Mulkidjanian et al., 2006; Sjöholm et al., 2017; Morelli et al., 2019; Kell 2021, 2024; Nesterov et al., 2024; Variyam et al., 2024). Most of the modified chemiosmotic models suggest that protons, instead of being released to the bulk aqueous phase, are conducted along the surface of membranes towards the ATP synthase. As pointed out by Goñi (2014), Mitchell's chemiosmotic concept can be reconciled with these models, if the value of pmf is „calculated surface‐to‐surface, rather than bulk‐to‐bulk”; which is in line with the conclusion by Kell (1979) that “the functional proton current under normal conditions is confined to the interphase regions”.

The ‘proton‐conduction’ models are strongly supported by the discovery that water molecules at the membrane‐water interface represent an electrostatic barrier toward the bulk water phase (Mulkidjanian et al., 2006). Water‐protein and water‐membrane interfaces in general, are comprised of polarized multilayers of water molecules, aka layers of confined water, which possess distinct structural and dynamical features compared to bulk water (Dér et al., 2007; Ghosh et al., 2007; Nagata and Mukamel 2010; Trapp et al., 2010; Nickels and Katsaras 2015; Frias and Disalvo 2021; Bellissent‐Funel 2023). Proton‐binding networks, or so‐called ‘proton antennas’, which can be formed, e.g., by clusters of H‐bond‐forming (carboxylic) side chains of membrane proteins or lipid head groups and water molecules have been proposed to exist in different biological systems such as PSII, channel rhodopsins and GFP complexes (Shinobu et al., 2010; Bondar 2022; Aoyama et al., 2024). Proton antennas are able to gather and transiently store protons.

According to a recently proposed mechanism, the so‐called “protet mechanism (Kell 2021), energy coupling in oxidative and photosynthetic phosphorylation, is based on protein networks. The basic assumption of the ‘protet’ model, or protonic charge transfer model, is that protons are stored in and drained from protein networks (Kell 2021, 2024). When applying the ‘protet’ model for the energization of TMs and the utilization of pmf, we also invoke the role of bound water molecules and assume that protons – produced upon splitting water at the donor side of PSII, and ejected upon the oxidation of plastoquinol molecules by the cyt *b*_6_ *f* complex – are not released to the bulk water phase of the lumen; instead, they remain in the hydration layer of the proteins. By this means, protons can be accumulated on protein residues on the lumenal side. Their release into the lumen, which is densely packed with a large variety of proteins could easily be dispersed and ‘lost’ for ATP synthesis (Gollan et al., 2021; Farci and Schröder 2023). It is an interesting question, which deserves further considerations, what could be the spectral fingerprints of these transiently stored protons and how the proposed protet mechanism(s), the storage of protons (Kell 2024), is harmonized with known ΔpH‐dependent regulatory mechanisms (Ruban and Saccon 2022; Iwai et al., 2024).

For the conduction of protons for long distances from PSII to the ATP synthase, we invoke the Grotthus mechanism, or proton jumping mechanism. According to this mechanism, the deposited protons e.g., generated by PSII, diffuse through the hydrogen bond network of water molecules (Agmon 1995). The feasibility of long‐distance proton migration has recently been demonstrated on self‐assembled peptide constructs, which allow proton transport over micrometer‐size distances (Censor et al., 2024).

Using the above premises, we hypothesize that the integrity of the protein network, which allows the efficient generation and utilization of pmf in TMs, is secured by the high abundance of MGDG. To prevent the disruption of this network via ‘diluting’ the protein density of TMs by lipids, MGDG must be present in high amounts relative to the bilayer lipids. It appears that other roles of MGDG as non‐bilayer lipids, such as supporting the activity of VDE and mediating membrane fusions, require much lower concentrations. Indeed, high de‐epoxidation rates were obtained with MGDG concentrations that were significantly lower than in TMs (Latowski et al., 2002). Also, fusion of most biological membranes occurs at much lower concentrations of non‐bilayer lipids. In contrast, the segregation capability of lipids in excess depends prominently on the concentration of non‐bilayer lipids, as reflected by the strong dependence of the formation of the H_II_ phase on the concentration of MGDG (Fehér et al., 2025). In general, the proportion of non‐bilayer lipids in lipid mixtures largely determines their non‐bilayer propensity (Seddon and Templer 1995; Williams 1998). This, in turn, adjusts the protein‐to‐lipid ratio of the membrane (Garab et al., 2000).

With regard to the role of water in TMs, recent experimental findings have indicated that ordered layers of water molecules play a crucial role in maintaining the ultrastructure of chloroplasts (Zsíros et al., 2020). They showed that water‐structure‐breaking salts, such as sodium perchlorate or sodium thiocyanate, led to a rapid disassembly of the TM ultrastructure. Numerous data also indicate that lipid head groups are tightly associated with water (Trapp et al., 2010; Nickels and Katsaras 2015). It is equally important to realize that in the presence of non‐bilayer lipids, the polymorphic lipid phase behavior of membranes is highly sensitive to their hydration state (Seddon and Templer 1995; van Eerden et al., 2015; Fehér et al., 2024). Hence, this factor adds another dimension to the structural dynamics of TMs – strongly suggesting that the high abundance of MGDG has important physiological / regulatory roles. This notion is supported by the facts that the MGDG‐deficient *Arabidopsis thaliana* mutant *mgd1* suffers from defects in chloroplast ultrastructure (Jarvis et al., 2000) and remodelling of glycerolipids plays an important role in stress physiology (Yu et al., 2021).

**FIGURE 6:**
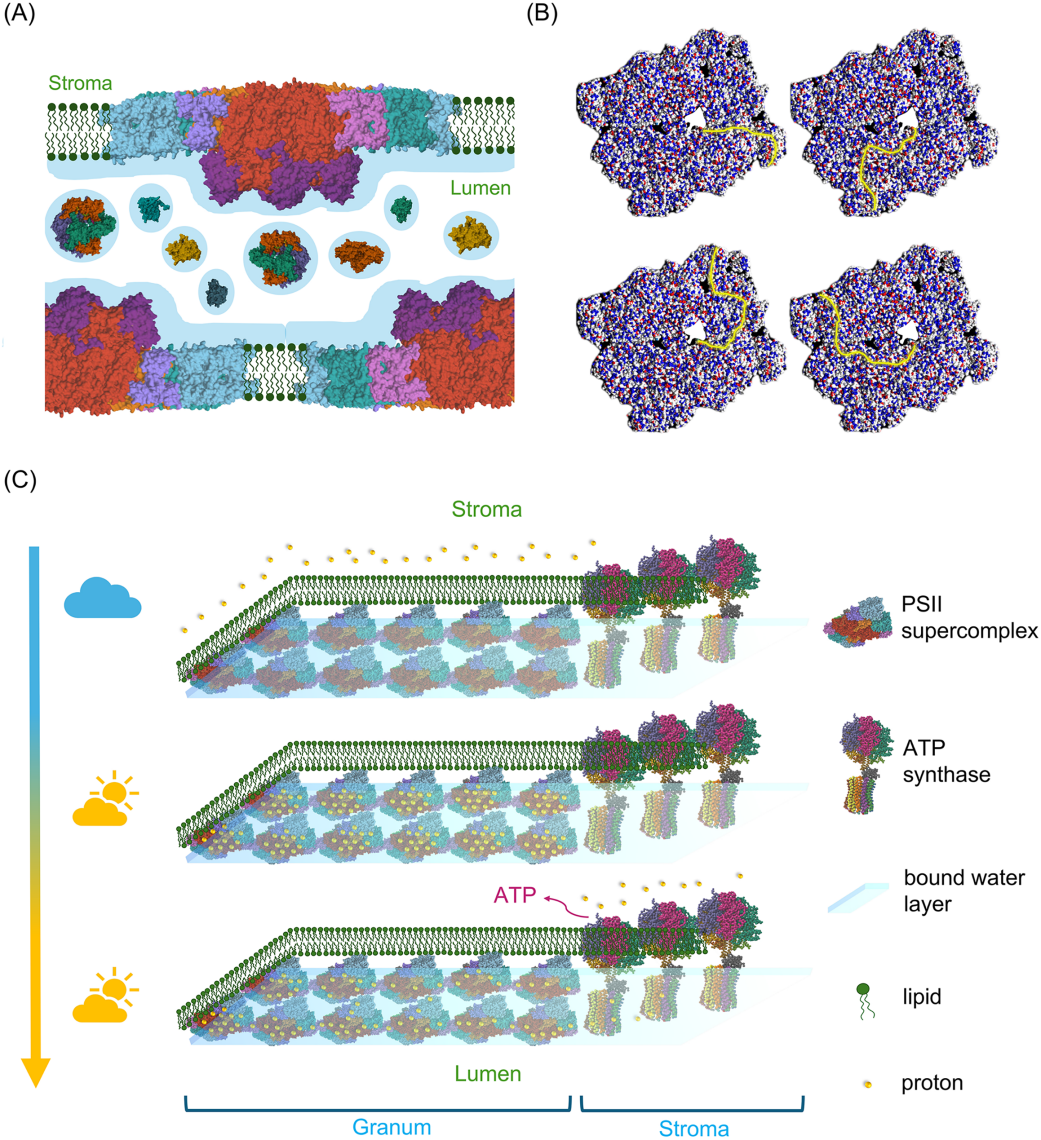
Schematic model, illustrating the main elements of the proposed modified chemiosmotic mechanism of ATP synthesis in plant TMs. Here we use tenets of the ‘protet’ model (Kell 2024), according to which protons in energy converting membranes are stored in and drained from protein networks for ATP synthesis. Our model heavily relies on the high abundance of MGDG, which – via lending the capability of segregating ‚excess' lipids from the bilayer – warrants the formation and stability of high‐density protein networks in TMs (Garab et al., 2000). Further, we assume that „the membrane surface is separated from the bulk aqueous phase by a barrier of electrostatic nature” (Mulkidjanian et al., 2006), which thus facilitates the conduction of protons along the membrane surface. This prevents the binding of protons to proteins in the lumen, which contains at least 78 different proteins (Farci and Schröder 2023). Panel (A) illustrates that PSII‐LHCII supercomplexes are embedded in the lipid bilayer and that the lumen contains different water‐soluble proteins (selected PDBs: 3QO6, 1FC6, 5X56). Panel (B) shows the distribution of positive (red) and negative (blue) charges on the lumenal side of the supercomplex; yellow lines depict some of the different possible proton‐conduction pathways (for an animation, see Supplementary Movie 1). Panel (C) depicts the proposed series of events: in the dark, the protons (red dots) are located on the stroma side. Upon illumination, they are deposited on the lumenal surface of supercomplexes (here only PSII‐LHCII supercomplexes are displayed). Finally, the activation of ATP synthase (PDB: 1QO1) drains the protons from these sites and the protons ‚diffuse’ from the grana towards the stroma lamellae by Grotthus mechanism (Agmon 1995). This series of events is animated in Supplementary Movie 2.

In the light of the above findings, delocalization of Δμ_H_^+^ in the bulk aqueous phases seems unlikely, or at least substantially delayed compared to the proton‐conduction pathways along the membrane surfaces (Kell 2021, 2024). Key elements of the proposed mechanism of diffusion of PSII protons from the core of the grana to the ATP synthase in the stroma are displayed in Figure 6. We emphasize that the structural basis of this mechanism is provided by densely packed arrays of PSII‐LHCII supercomplexes, warranted by the high abundance of MGDG. This quasi‐continuous protein network possesses protonable residues at the lumenal side.

## 6 CONCLUDING REMARKS

We reviewed experimentally established facts on the polymorphic phase behavior of plant TMs and stressed the fundamental significance of non‐bilayer lipid phases in the self‐assembly and structural and functional dynamics of TMs. We hypothesize that non‐bilayer lipid phases facilitate the formation of functional sub‐compartments in plant TMs, allowing an energetic autonomy of each granum stroma TM assembly. Further, it is pointed out that the compromised electrical insulation capability of TMs, due to the presence of MGDG, is not necessarily in conflict with the generation of pmf and its utilization for ATP synthesis – if using a modified chemiosmotic theory. In fact, the high abundance of MGDG might well be a key boundary condition that secures the operation of the energy converting machinery, which is proposed to be based on proton‐conduction pathways on the quasi‐continuous, hydrated protonable protein residues on the lumenal side of TMs.

The strong dependence of the phase behavior of lipid mixtures on their hydration state and the sensitivity of the lipid polymorphism of TMs to factors, such as the ΔpH, ionic and the osmotic strength of the stroma liquid, as well as the ambient temperature strongly suggest the involvement of non‐bilayer lipid phases in regulatory mechanisms governing e.g. TM responses to heat and drought stresses of plants.

## AUTHOR CONTRIBUTIONS

The concepts of the study were conceived by G.G. and V.Š. and were gradually refined with the participation of all authors. K.B., O.D. and V.K. focused on aspects of the polymorphic lipid phase behavior of thylakoid membranes, and Z.N. and A.D. on the energization of energy‐converting membranes, in general. The figures were prepared by K.B. G.G. edited the drafts and the final version of the manuscript with the help of K.B. and O.D. All authors have read and approved the submitted manuscript.

## FUNDING INFORMATION

This work was supported by grants from the Czech Science Foundation (GAČR 23‐07744S to G.G.), the European Union under the LERCO project (CZ.10.03.01/00/22_003/0000003) via the Operational Programme Just Transition, and the Hungarian National Research Excellence Programme (NKFI‐1 150958 to A.D.).

## Supporting information


**Legend to Supplementary Movie 1**: Spontaneously formed pathways of protons on the lumenal side of PSII‐LHCII supercomplexes. For further details, see the legend to Figure 6b.
**Legend to Supplementary Movie 2**: The formation of pmf and its utilization for ATP synthesis in plant TMs – for details of this hypothesis, see Legend to Figure 6.

## Data Availability

Data sharing is not applicable to this article as no new data were created or analyzed in this study.
